# Smac/DIABLO expression in human gastrointestinal carcinoma: Association with clinicopathological parameters and survivin expression

**DOI:** 10.3892/ol.2014.2598

**Published:** 2014-10-09

**Authors:** MICHIKO SHINTANI, AKIKO SANGAWA, NAOKI YAMAO, SHINGO KAMOSHIDA

**Affiliations:** 1 Laboratory of Pathology, Division of Medical Biophysics, Kobe University Graduate School of Health Sciences, Kobe, Hyogo 654-0142, Japan; 2 Department of Diagnostic Pathology, Osaka Red Cross Hospital, Osaka 543-8555, Japan; 3 Department of Clinical Laboratory, Kuma Hospital, Kobe, Hyogo 650-0011, Japan

**Keywords:** survivin, Smac/DIABLO, immunohistochemistry, apoptosis

## Abstract

Lack of apoptosis is a key factor in carcinogenesis and tumor progression. Survivin is a member of the inhibitor of apoptosis protein (IAP) family. Second mitochondria-derived activator of caspases/direct inhibitor of apoptosis-binding protein with low pI (Smac/DIABLO) is an antagonist of IAPs. Recently, Smac/DIABLO was identified as a potent therapeutic target. However, the clinical significance of Smac/DIABLO in gastrointestinal carcinomas remains unclear. In the present study, Smac/DIABLO expression was analyzed by immunohistochemistry in 72 gastric adenocarcinomas and 78 colorectal adenocarcinomas. The expression of Smac/DIABLO was significantly higher in colorectal carcinoma than in gastric carcinoma. Additionally, a correlation was found between the expression of Smac/DIABLO and nuclear survivin in well- to moderately-differentiated colorectal adenocarcinomas (r=0.245; P<0.01). Based on these results, it was hypothesized that gastric and colorectal carcinomas differ in the level of Smac/DIABLO expression. Our previous studies revealed that the expression of cleaved caspase-9 was significantly lower in colorectal carcinoma than in gastric carcinoma (P<0.0001). Conversely, the expression levels of microtubule-associated protein 1 light chain 3 (LC3), an autophagy marker, and survivin were significantly higher in colon cancer than in gastric cancer (P<0.0001 and P<0.01, respectively). Taken together, these results indicate that not only LC3 and survivin expression, but also Smac/DIABLO expression, are significantly higher in colorectal carcinoma than in gastric carcinoma. We hypothesize that the analysis of Smac/DIABLO, survivin and LC3 expression in colorectal carcinoma is likely to aid cancer therapy due to the involvement of these markers in apoptosis and/or autophagy.

## Introduction

Second mitochondria-derived activator of caspases/direct inhibitor of apoptosis-binding protein with low pI (Smac/DIABLO) is a mitochondrial apoptogenic molecule that is released from the mitochondria in response to apoptotic stress. Smac/DIABLO is known to antagonize the function of inhibitors of apoptosis proteins (IAPs), including X-linked inhibitor of apoptosis (XIAP), cellular IAP1/2 and survivin (1–7). The lack of Smac/DIABLO expression may inhibit apoptosis in cancer cells, promoting survival. Previous studies have indicated that decreased levels of Smac/DIABLO correlate with cancer progression. Smac/DIABLO is considered a potent therapeutic target (8–11). However, the clinical significance of Smac/DIABLO in various cancers remains unclear.

Survivin, a member of the IAP family, exhibits a dual cellular function as an inhibitor of apoptosis and a regulator of mitosis. The anti-apoptotic effect of survivin is associated with the inhibition of caspase activity. Furthermore, survivin also acts as a chromosome passenger protein, regulating the G_2_ and M phases of the cell cycle (12–17). High levels of survivin expression are found in numerous embryonic tissues and in the majority of human tumors. By contrast, extremely low level or undetectable levels of expression are found in differentiated adult tissues. As a result, survivin may prove to be a marker for tumor progression and prognosis (18–20).

Apoptosis is a primary biochemical cell-death pathway that is critical for normal tissue homeostasis, cellular differentiation and development (21,22). Apoptosis is regulated by the activity of caspases via two possible pathways, the death receptor-mediated apoptotic pathway (extrinsic pathway, involving caspase 8) and the mitochondrial-mediated apoptotic pathway (intrinsic pathway, involving caspase 9) (21–23). Generally, IAPs suppress apoptosis via the inhibition of caspase activation.

The expression of cleaved caspase (CC)8 and 9, microtubule-associated protein 1 light chain 3 (LC3), an autophagy marker, and survivin in gastric and colorectal carcinomas has been investigated to elucidate the cell death pathway (24,25). Results from our previous studies revealed that the expression of CC9 in colorectal carcinoma was significantly lower than that in gastric carcinoma (P<0.0001) (24). By contrast, the expression of LC3 and survivin in colon carcinoma was significantly higher than that in gastric carcinoma (P<0.0001 and P<0.01, respectively) (24,25). These results indicated that different cellular death pathways are activated in gastric and colorectal carcinomas (24).

In the present study, the expression of Smac/DIABLO in gastric and colorectal carcinoma was examined using immunohistochemistry. Furthermore, the association between Smac/DIABLO expression and clinicopathological parameters was investigated. Additionally, the correlation between the expression of Smac/DIABLO and survivin was elucidated.

## Materials and methods

### Tissue samples

Surgically resected tumor tissues were collected from the archives of the Department of Diagnostic Pathology of the Osaka Red Cross Hospital (Osaka, Japan) and the Kobe Central Hospital of Social Insurance (Kobe, Japan). A total of 72 advanced gastric adenocarcinomas (36 well- to moderately-differentiated and 36 poorly-differentiated) and 78 colorectal adenocarcinomas (68 well- to moderately-differentiated and 10 poorly-differentiated) were analyzed for Smac/DIABLO expression by immunohistochemistry. The study was approved by the ethics committee of Kobe University Graduate School of Health Sciences (Kobe, Japan). The tumors were classified according to the tumor-node-metastasis (TNM) classification of malignant tumors (TNM 2009) (26). All specimens were preserved in 10% formalin and embedded in paraffin. Sections that were 3-μm thick were cut consecutively and mounted on aminopropyltriethoxysilane-coated slides.

### Immunohistochemical staining

Smac/DIABLO expression was analyzed using immunohistochemistry. The tissue sections were deparaffinized with xylene (Nacalai Tesque, Inc., Kyoto, Japan) and dehydrated using a graded series of ethanol solutions. Antigen retrieval was performed by immersing the slides in 10 mM citrate buffer (pH 7.0; Nacalai Tesque, Inc.) and heating for 10 min in a pressure cooker (Tefal, Haute-Savoie, France). The sections were then cooled at room temperature in a soaking solution (10 mM citrate buffer; pH 7.0; Nacalai Tesque, Inc.) for 30 min. Next, the sections were washed with water, followed by 10 mM phosphate-buffered saline (PBS; pH 7.2). Following blocking with 0.25% casein in PBS (Dako, Glostrup, Denmark), the sections were incubated with a mouse monoclonal antibody against Smac/DIABLO (1:1,000; Cell Signaling Technology, Inc., Danvers, USA) overnight at room temperature. The sections were then rinsed with PBS. To detect Smac/DIABLO, the sections were incubated with Histofine Simple Stain MAX-PO (Nichirei Bioscience Inc., Tokyo, Japan) for 1 h at room temperature. The reaction products were then developed using 3,3′-diaminobenzidine (Dako, Glostrup, Denmark) and counterstained using Mayer’s hematoxylin (Merck KGaA, Darmstadt, Germany). For the negative control, a section was treated as described above, but PBS was used instead of the primary antibody.

### Evaluation of immunostaining

Sections were considered positive for Smac/DIABLO if cytoplasmic staining was observed. A mean percentage of positive tumor cells was determined in ≥5 areas at a magnification of ×400 and assigned to the following categories: 0, negative; 1, <30%; 2, 30–69%; and 3, ≥70%. The intensity of Smac/DIABLO immunostaining was scored as follows: 1, weak; 2, moderate; and 3, intense. A final score was obtained by calculating the sum of these two scores. Cases with final scores of <5 were considered to exhibit low expression, whereas cases with final scores of ≥5 were considered to exhibit high expression.

### Statistical analysis

Differences in the expression of Smac/DIABLO between gastric and colorectal adenocarcinomas, well- to moderately-differentiated and poorly-differentiated adenocarcinomas, patient age and gender, as well as lymphatic and vascular invasion were evaluated using the χ^2^ and Fisher’s exact tests. The correlation between marker expression and tumor location, depth of invasion, lymph node metastasis and pathological stage were analyzed using the Kruskal-Wallis test. Spearman’s rank correlation was used to assess the correlation between the different markers. P<0.05 was considered to indicate a statistically significant difference.

## Results

### Expression of Smac/DIABLO in gastric and colorectal carcinomas

**Figure 1:**
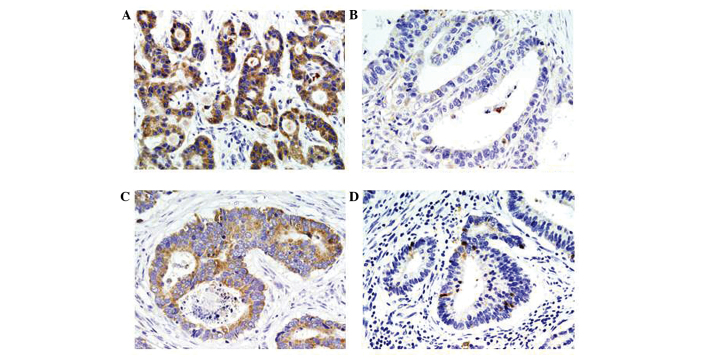
Immunhistochemical staining of Smac/DIABLO in gastrointestinal carcinomas. Smac/DIABLO immunoreactivity was predominantly observed in the cytoplasm. (A) High expression of Smac/DIABLO in gastric carcinoma. (B) Low expression of Smac/DIABLO in gastric carcinoma. (C) High expression of Smac/DIABLO in rectal carcinoma. (D) Low expression of Smac/DIABLO in rectal carcinoma. Smac/DIABLO, second mitochondria-derived activator of caspases/direct inhibitor of apoptosis-binding protein with low pI. (A–D, magnification, ×200).

Smac/DIABLO expression was predominantly observed in the cytoplasm (Fig. 1). Smac/DIABLO-positive staining was observed in 46% (33/72) of gastric carcinomas. Smac/DIABLO expression was higher in well- to moderately-differentiated specimens (50%) compared with poorly-differentiated specimens (42%), however, no significant differences were identified (Table I).

### Smac/DIABLO-positive staining

Smac/DIABLO-positive staining was observed in 69% (54/78) of colorectal carcinomas. The expression of Smac/DIABLO was significantly higher in colorectal carcinoma than in gastric carcinoma (P<0.01). Smac/DIABLO expression was higher in well- to moderately-differentiated specimens (71%) compared with poorly-differentiated specimens (60%), however, no significant differences were identified.

### Correlation between Smac/DIABLO expression and clinicopathological parameters

Tables II and III present the associations between Smac/DIABLO protein expression and the clinicopathological parameters. In gastric carcinomas, none of the parameters of patient age, gender, tumor location, depth of invasion, lymph-node metastasis, lymphatic invasion, vascular invasion or pathological stage were found to be associated with the positive expression of Smac/DIABLO. In colorectal carcinomas, the level of Smac/DIABLO expression was significantly higher in cases without vascular invasion than in the cases with vascular invasion (P<0.05). However, none of the other parameters of patient age, gender, tumor location, depth of invasion, lymph-node metastasis, lymphatic invasion, or pathological stage were found to be associated with positive Smac/DIABLO expression. Although no significant difference was identified between the majority of the clinicopathological parameters and the expression of Smac/DIABLO, a trend towards an association between a decreased expression level of Smac/DIABLO and tumor stage was identified.

### Association between Smac/DIABLO expression in gastric and colorectal carcinomas

A correlation was identified between Smac/DIABLO and nuclear survivin (r=0.245; P<0.01) in well- to moderately-differentiated colorectal adenocarcinomas. However, in all cases, the expression of Smac/DIABLO was not significantly associated with survivin expression (P=0.08).

## Discussion

In the present study, the expression of Smac/DIABLO in gastric and colorectal carcinomas was investigated and compared by immunohistochemistry. Smac/DIABLO-positive staining was observed in 46% (33/72) and 69% (54/78) of gastric and colorectal carcinomas, respectively. These results revealed that the expression of Smac/DIABLO was significantly higher in colorectal carcinoma than in gastric carcinoma (P<0.01). Regarding the association between Smac/DIABLO expression and tumor type, Yoo *et al* (8) analyzed archival tissues of 100 carcinomas and 50 sarcomas from various origins using immunohistochemistry. The study reported that Smac/DIABLO was differentially expressed among cancer types and indicated that gastric, colorectal and ovarian carcinomas exhibited a high frequency of Smac/DIABLO expression, whereas Smac/DIABLO expression in prostate carcinoma and non-small cell lung carcinoma was low. According to previous studies, the positive rates of expression in gastric and colorectal carcinomas are 13–70% and 66–90%, respectively (9,10,27–29). Taken together, these studies indicate that Smac/DIABLO expression in colorectal carcinoma is higher than that in gastric carcinoma, however, differences may be observed due to the different counting methods used.

The results of the present study demonstrated that Smac/DIABLO expression in well- to moderately-differentiated gastric and colorectal carcinomas was higher than that in poorly-differentiated adenocarcinomas. However, no statistically significant difference was identified. Kim *et al* (27) reported that Smac/DIABLO expression was associated with a higher proportion of diffuse histology types than overall cases (intestinal, diffuse and mixed types). By contrast, Shibata *et al* (28) identified a significant correlation between Smac/DIABLO expression and tumor differentiation (P<0.0001), whereby patients with high Smac/DIABLO expression presented more differentiated tumors. Yoo *et al* (8) identified no correlation between histological subtype (diffuse type vs. intestinal type) and the expression of Smac/DIABLO (8). Based on these results, we hypothesized that there is no association between the expression of Smac/DIABLO and tumor differentiation in gastric and colorectal carcinomas.

Furthermore, the association between Smac/DIABLO expression and clinicopathological parameters was investigated in the present study. In colorectal carcinomas, the level of Smac/DIABLO expression was significantly higher in the cases without vascular invasion than in the cases with vascular invasion (P<0.05). However, this association was not identified in gastric carcinoma. Previous studies have demonstrated that Smac/DIABLO expression is associated with a good prognosis and low tumor stage (8–11). In the present study, a trend towards an association between decreased Smac/DIABLO expression and pathological stage in gastric and colorectal carcinomas was observed, however, no statistically significant difference was identified. Endo *et al* (9) reported that no significant difference was present between the two Smac/DIABLO-positive and -negative tumor groups with respect to tumor size, tumor location, histological differentiation and lymphatic and venous invasion. The clinical significance of Smac/DIABLO expression in various cancers remains unclear. Therefore, additional comprehensive studies are required to elucidate the clinical significance of Smac/DIABLO expression in gastric and colorectal carcinomas.

In the present study, the expression of Smac/DIABLO and nuclear survivin were found to correlate in well- to moderately-differentiated colorectal adenocarcinomas (r=0.245; P<0.01). De Oliveira Lima *et al* (30) identified a correlation between the expression of survivin and Smac/DIABLO in colorectal carcinoma using immunohistochemistry. The results of the present study only identified a correlation in well- to moderately-differentiated cases, however, the correlation was not observed in all colorectal cancer cases. By contrast, Kim *et al* (27) revealed that Smac/DIABLO expression was not associated with survivin, whereas Smac/DIABLO expression was found to inversely correlate with the expression of XIAP, an IAP, using immunohistochemistry. To the best of our knowledge, only a small number of studies have demonstrated the association between Smac/DIABLO and survivin expression using immunohistochemistry. Endo *et al* (9) proposed that the decrease of Smac/DIABLO expression is an independent factor of poor prognosis for colorectal cancer patients, while other studies have indicated that survivin may be a marker for tumor progression and prognosis (5,31,32). Survivin is found in the nucleus and cytoplasm. Nuclear survivin is considered a promoter of cell proliferation, whereas cytoplasmic survivin is considered to exhibit cytoprotective effects (16,33), however, the mechanisms involved remain unclear. Previous studies have indicated that nuclear and cytoplasmic survivin expression is associated with an improved prognosis in gastric carcinomas. By contrast, in colorectal carcinoma, the upregulation of cytoplasmic survivin is associated with a poor prognosis (31,32,34–37). However, there is no disagreement between the results of previous studies and the present study. At present, the association between survivin or Smac/DIABLO and the clinical prognosis remains controversial. Thus, further studies are required to confirm the association between Smac/DIABLO expression and nuclear or cytoplasmic survivin expression.

In conclusion, the present study demonstrated that the expression of Smac/DIABLO was significantly higher in colorectal carcinoma than in gastric carcinoma. Additionally, a correlation was found between the expression of Smac/DIABLO and nuclear survivin in well- to moderately-differentiated colorectal adenocarcinomas (r=0.245; P<0.01). Based on these results, we hypothesized that gastric and colorectal carcinomas differ in their levels of Smac/DIABLO expression. These results, in addition to our previous results, indicate that not only LC3 and survivin expression levels, but also Smac/DIABLO expression levels, are significantly higher in colorectal carcinoma than in gastric carcinoma (24,25). We hypothesized that the expression of Smac/DIABLO in colorectal carcinoma may be upregulated to suppress the anti-apoptotic effect of survivin. Furthermore, the results of this study indicate that the analysis of Smac/DIABLO, survivin and LC3 expression in colorectal carcinoma is likely to aid cancer therapy due to the involvement of these markers in apoptosis and/or autophagy.

## Figures and Tables

**Table I:** Expression of Smac/DIABLO in gastrointestinal adenocarcinomas.

Adenocarcinoma type	Total, n	High expression, n (%)	Low expression, n (%)
Gastric adenocarcinoma	72	33 (46)^a^	39 (54)
Well- to moderately-differentiated	36	18 (50)	18 (50)
Poorly-differentiated	36	15 (42)	21 (58)
Colorectal adenocarcinoma	78	54 (69)^a^	24 (31)
Well- to moderately-differentiated	68	48 (71)	20 (29)
Poorly-differentiated	10	6 (60)	4 (40)

a P<0.01, gastric adenocarcinoma vs. colotrectal adenocarcinoma.

Smac/DIABLO, second mitochondria-derived activator of caspases/direct inhibitor of apoptosis-binding protein with low pI.

**Table II:** Correlation between Smac/DIABLO expression and clinicopathological parameters in gastric adenocarcinomas.

Clinicopathological parameters	Total, n	Smac/DIABLO expression, n (%)	

		High	Low
Age, years			
<60	10	4 (40)	6 (60)
>60	62	29 (47)	33 (53)
Gender			
Male	51	23 (45)	28 (55)
Female	21	10 (48)	11 (52)
Location			
Cardia	17	7 (41)	10 (59)
Fundus	31	15 (48)	16 (52)
Antrum	24	11 (46)	13 (54)
Depth of invasion			
pT2	17	11 (65)	6 (35)
pT3	20	9 (45)	11 (55)
pT4	35	13 (37)	22 (63)
Lymph node metastasis			
pN0	23	14 (61)	9 (39)
pN1	15	4 (27)	11 (73)
pN2	11	4 (36)	7 (64)
pN3	23	11 (48)	12 (52)
Lymphatic invasion			
Negative	16	7 (44)	9 (56)
Positive	56	26 (46)	30 (54)
Vascular invasion			
Negative	38	14 (37)	24 (63)
Positive	34	19 (56)	15 (44)
UICC p-Stage			
IB	11	8 (73)	3 (27)
IIA and IIB	22	9 (41)	13 (59)
IIIA, IIIB and IIIC	39	16 (41)	23 (59)

UICC, Union for International Cancer Control; Smac/DIABLO, second mitochondria-derived activator of caspases/direct inhibitor of apoptosis-binding protein with low pI.

**Table III:** Correlation between Smac/DIABLO expression and clinicopathological parameters in colorectal adenocarcinomas.

Clinicopathological parameters	Total, n	Smac/DIABLO expression, n (%)	

		High	Low
Age, years			
<60	22	16 (73)	6 (27)
>60	56	38 (68)	18 (32)
Gender			
Male	44	29 (66)	15 (34)
Female	34	25 (74)	9 (26)
Location			
Right colon	24	16 (67)	8 (33)
Left colon	24	19 (79)	5 (21)
Rectum	30	19 (63)	11 (37)
Depth of invasion			
pT2	7	5 (71)	2 (29)
pT3	46	31 (67)	15 (33)
pT4	25	18 (72)	7 (28)
Lymph node metastasis			
pN0	35	25 (71)	10 (29)
pN1	31	23 (74)	8 (26)
pN2	12	6 (50)	6 (50)
Lymphatic invasion			
Negative	24	16 (67)	8 (33)
Positive	54	38 (70)	16 (30)
Vascular invasion			
Negative	40	32 (80)^a^	8 (20)
Positive	38	22 (58)^a^	16 (42)
UICC p-Stage			
IB	6	5 (83)	1 (17)
IIA and IIB	28	20 (71)	8 (29)
IIIA, IIIB and IIIC	44	29 (66)	15 (34)

a P<0.05, negative vascular invasion vs. positive vascular invasion.

UICC, Union for International Cancer Control; Smac/DIABLO, second mitochondria-derived activator of caspases/direct inhibitor of apoptosis-binding protein with low pI.
